# Hypoxia-Induced Epithelial-Mesenchymal Transition Is Involved in Bleomycin-Induced Lung Fibrosis

**DOI:** 10.1155/2015/232791

**Published:** 2015-12-27

**Authors:** Liang Guo, Jun-mei Xu, Lei Liu, Su-mei Liu, Rong Zhu

**Affiliations:** ^1^Department of Anesthesiology, Shandong Provincial Qianfoshan Hospital, Shandong University, Jinan, Shandong 250014, China; ^2^Department of Anesthesiology, Second Xiangya Hospital, Central South University, Changsha, Hunan 410011, China

## Abstract

Pulmonary fibrosis is a severe disease that contributes to the morbidity and mortality of a number of lung diseases. However, the molecular and cellular mechanisms leading to lung fibrosis are poorly understood. This study investigated the roles of epithelial-mesenchymal transition (EMT) and the associated molecular mechanisms in bleomycin-induced lung fibrosis. The bleomycin-induced fibrosis animal model was established by intratracheal injection of a single dose of bleomycin. Protein expression was measured by Western blot, immunohistochemistry, and immunofluorescence. Typical lesions of lung fibrosis were observed 1 week after bleomycin injection. A progressive increase in MMP-2, S100A4, *α*-SMA, HIF-1*α*, ZEB1, CD44, phospho-p44/42 (p-p44/42), and phospho-p38 MAPK (p-p38) protein levels as well as activation of EMT was observed in the lung tissues of bleomycin mice. Hypoxia increased HIF-1*α* and ZEB1 expression and activated EMT in H358 cells. Also, continuous incubation of cells under mild hypoxic conditions increased CD44, p-p44/42, and p-p38 protein levels in H358 cells, which correlated with the increase in S100A4 expression. In conclusion, bleomycin induces progressive lung fibrosis, which may be associated with activation of EMT. The fibrosis-induced hypoxia may further activate EMT in distal alveoli through a hypoxia-HIF-1*α*-ZEB1 pathway and promote the differentiation of lung epithelial cells into fibroblasts through phosphorylation of p38 MAPK and Erk1/2 proteins.

## 1. Introduction

Pulmonary fibrosis is a severe and crippling disease that contributes to the morbidity and mortality of a number of pediatric and adult lung diseases. Fibrosis is a common response to a variety of physical, chemical, and biological injuries, such as viral infection [1], autoimmune reaction [2], mineral dusts [3], radiation [4], and some medications [5]. Although variations in the pathologic characteristics of pulmonary fibrosis are etiologically dependent, a number of characteristics, including inflammatory cell infiltration, mesenchymal cell proliferation, and excessive synthesis of extracellular matrix (ECM) components [6, 7], are shared among various types of pulmonary fibrosis.

Excessive synthesis of extracellular matrix (ECM) components is a prominent feature of fibrosis, and ECM degradation is controlled primarily by matrix metalloproteinases (MMPs), a family of secreted, zinc-dependent enzymes. MMP-2 protein level has been previously reported to be increased in the lung tissues of bleomycin-treated animals and is preferentially secreted by fibroblasts and epithelial cells [8]. Fibroblast-specific protein 1 (FSP1), also known as S100A4, is a cytoplasmic calcium-binding protein and a marker for fibroblasts of mesenchymal origin [9]. Alpha-smooth muscle actin (*α*-SMA) is a marker of myofibroblasts, and a previous study demonstrated that *α*-SMA was significantly elevated in bleomycin-induced pulmonary fibrosis [10]. Therefore, changes in MMP-2, S100A4, and *α*-SMA levels could reflect the activation of the fibrosis process.

The accumulated evidence has demonstrated that epithelial cells can participate in the repair of lung injury through a process called epithelial-mesenchymal transition (EMT) [11]. A number of previous studies have provided evidence of EMT in pathological tissue fibrosis and suggested that fibroblasts/myofibroblasts in models of lung fibrosis may be directly derived from epithelial cells [12]. A previous study in an animal model demonstrated that approximately one-third of the S100A4-positive fibroblasts were derived from lung epithelium 2 weeks after bleomycin administration, suggesting that EMT contributes to bleomycin-induced lung fibrosis [13]. However, the molecular mechanisms driving the EMT process and further progression of fibrosis have not been fully elucidated. CD44 is a marker of mesenchymal stem cells, including cells that acquire the ability to differentiate from the EMT process [14]. Previous studies demonstrated that ROS-induced differentiation of embryonic stem cells is associated with enhanced Erk1/2 phosphorylation [15]. Inhibitors of p38 MAPK were found to reduce radiation-induced fibroblast differentiation [16]. However, whether Erk1/2 and p38 MAPK were involved in the activation of mesenchymal stem cells and subsequent bleomycin-induced lung fibrosis have not been reported.

The lung is the conduit for oxygen uptake. Therefore, septal thickening during fibrosis will result in decreased blood and tissue oxygenation [17]. Hypoxia is the main pathological characteristic of pulmonary fibrosis, and lung tissues are highly sensitive to hypoxia. A previous study demonstrated that hypoxia induced EMT in alveolar epithelial cells through activation of mitochondrial HIF and endogenous transforming growth factor-*β*1 (TGF-*β*1) signaling [18]. Joseph et al. study revealed that a HIF-1*α*-ZEB1 signaling axis may promote hypoxia-induced EMT and invasion in glioblastoma [19]. Zhang et al. study showed that HIF-1*α*, but not HIF-2*α*, enhanced EMT and cancer metastasis in colorectal cancer cells by binding to the ZEB1 promoter [20]. However, whether the hypoxia-HIF-1*α*-ZEB1-EMT pathway is involved in pulmonary fibrosis has not been reported.

In this study, we investigated the dynamic changes of EMT in an animal model of pulmonary fibrosis, which was induced by intratracheal administration of bleomycin. We then further explored the molecular mechanisms associated with activation of EMT in cultured pneumocytes under hypoxic conditions.

## 2. Materials and Methods

### 2.1. Cell Culture

NCI-H358, a human lung type II epithelial cell line, and Beas-2B, a human bronchial epithelial cell line, were obtained from the American Type Culture Collection (ATCC). H358 cells were cultured in RPMI-1640 medium with 10% fetal bovine serum (FBS). Beas-2B cells were cultured in BEGM medium containing epithelial cell growth factors. Cells were cultured at 37°C, 5% CO_2_.

### 2.2. Animals

C57BL/6J male mice, weighing 25–30 g, were provided by the Animal Center of Central South University. All procedures involving animals were approved by the Institutional Committee of Animal Care, Central South University. All animal experiments were performed in accordance with the animal care guidelines of the Chinese Council. Mice were housed under a 12 h light/dark cycle with free water access and chow provided* ad libitum*.

### 2.3. Bleomycin Model

Bleomycin was prepared by dissolving bleomycin sulfate powder (Teva Parenteral Medicines, Irvine, CA) in sterile 0.9% saline. Bleomycin was injected intratracheally via intubation at a single dose of 0.06 mg in 0.1 mL saline solution per animal. Control animals received 0.1 mL of saline alone. For this procedure, mice were anesthetized with isoflurane by inhalation. At weeks 1, 3, and 6 following intratracheal bleomycin or saline instillation, animals were killed by an overdose of pentobarbital (80 mg/kg, i.p.). The lungs were excised. The right lungs were snap-frozen for Western blot analysis, while the left lungs were immersed and fixed in 4% paraformaldehyde in 0.1 M phosphate buffer for morphological analysis.

### 2.4. Morphological Examination

The fixed lung tissues were embedded in paraffin, sequentially sectioned into 4–6 *μ*m sections, and then stained with haematoxylin-eosin (H&E) solution and modified Masson's trichrome solution to detect the presence of collagen. The selected sections were viewed and photographed using a microscope. A minimum of 4 randomly selected fields were captured at 100x magnification for each section for evaluating fibrotic lung injury. Fibrosis was quantified using a modified Ashcroft scale [21]. Briefly, the fibrosis was graded from 0 to 8. Grades 1 to 3: The alveoli were partly enlarged and rarefied, while fibrotic changes gradually increased. Grade 4: Fibrotic masses appeared. Grade 5: Single fibrotic masses become confluent and lung structure is severely damaged starting from this stage. Grade 6: Variable alveolar septa are mostly nonexistent. Grade 7: Lung structure is mostly disorganized and alveoli become partially replaced with fibrotic masses. Grade 8: Alveoli become complete occlusions. Fibrosis in each lobe section was evaluated by two blinded observers. Two different methods were used to select the areas of evaluation: the most damaged part of the section and a random sampling. The final score represented an average of the two methods and all lobes.

### 2.5. Immunofluorescence

To perform immunofluorescence in lung tissues, the snap-frozen lungs were cut into 10–12 *μ*m sections and adhered to Poly-L-Lysine coated slides. The sections were fixed in 4% paraformaldehyde in PBS for 45 minutes and then blocked with 10% goat serum, followed by incubation with rabbit anti-mouse E-cadherin or rabbit anti-mouse vimentin antibody (Cell Signaling Technology, Beverley, MA) overnight at 4°C. Omission of the primary antibody served as negative control. FITC-labeled donkey anti-rabbit secondary antibodies (Jackson Immunoresearch) were used where relevant. The sections were mounted with mounting solution containing Dapi. To perform immunofluorescence in cultured cells, cells on slides were directly fixed with 4% paraformaldehyde and were then subjected to the procedure described above.

### 2.6. Immunohistochemistry

Four-micrometer-thick sections were cut from routinely paraffin-embedded lung tissues. The staining of CD44 was carried out according to the manufacturer's protocol. The deparaffinized sections were incubated with anti-mouse CD44 antibody (Abcam, Cambridge, MA, USA) (1 : 100 dilution) for 1 hour, followed by incubation with secondary antibody (Abcam) for 30 min. Substrate was added to the sections for 30 minutes followed by DAB staining and hematoxylin counterstaining. Positive staining was determined mainly by a brownish color in the cytoplasm of the cells. Omission of the primary antibody served as negative control.

### 2.7. Cell Hypoxia

H358 and Beas-2B cells at 70% confluency in 10 cm dishes were cultured under normoxic (21% O_2_) and hypoxic (2% O_2_) conditions for 12 h and 24 h. Cells were harvested for Western blot analysis. H358 and Beas-2B cells cultured under hypoxic (2% O_2_) conditions for 24 h were subsequently cultured under a mild hypoxic condition (10% O_2_) for 5 days. Cells were harvested for Western blot and immunofluorescence analysis.

### 2.8. Western Blot

Lung tissues or cultured cells were homogenized and Western blot was performed as previously described [22]. Briefly, 20–30 *μ*g of total protein was separated on 10% SDS-polyacrylamide gel electrophoresis and transferred onto polyvinylidene difluoride membranes (Immobilon-P; Millipore Corporation, Billerica, MA). The membranes were then blocked with 5% nonfat milk in PBST (PBS + 0.1% Tween-20) for 20 min and incubated with primary antibody overnight at 4°C. After washing with PBST, the membranes were probed with horseradish peroxidase-conjugated secondary antibody for 2 hrs. Protein bands were visualized with the ECL substrate (Millipore) and scanned using Photoshop. The densitometry unit of the protein expression was normalized to GAPDH levels. The antibodies for HIF-1*α*, E-cadherin, N-cadherin, vimentin, *α*-SMA, phospho-p44/42, total p44/42, phospho-p38, and total p38 MAPK were purchased from Cell Signaling Technology (Beverley, MA). Fibronectin and ZEB1 antibody were purchased from Santa Cruz Biotechnology (Santa Cruz, CA). CD44 antibody was purchased from Abcam (Cambridge, MA, USA).

### 2.9. Statistical Analysis

Data were analyzed using SPSS 16.0 (Statistical Package for the Social Science Version 16.0) and presented as the mean ± SEM. Analysis of differences between the two groups was performed using one-way analysis of variance (ANOVA) with* post hoc* Tukey test. Differences were considered significant at *P* < 0.05. 

## 3. Results

### 3.1. Bleomycin Induces Lung Fibrosis through Activation of MMP-2

**Figure 1:**
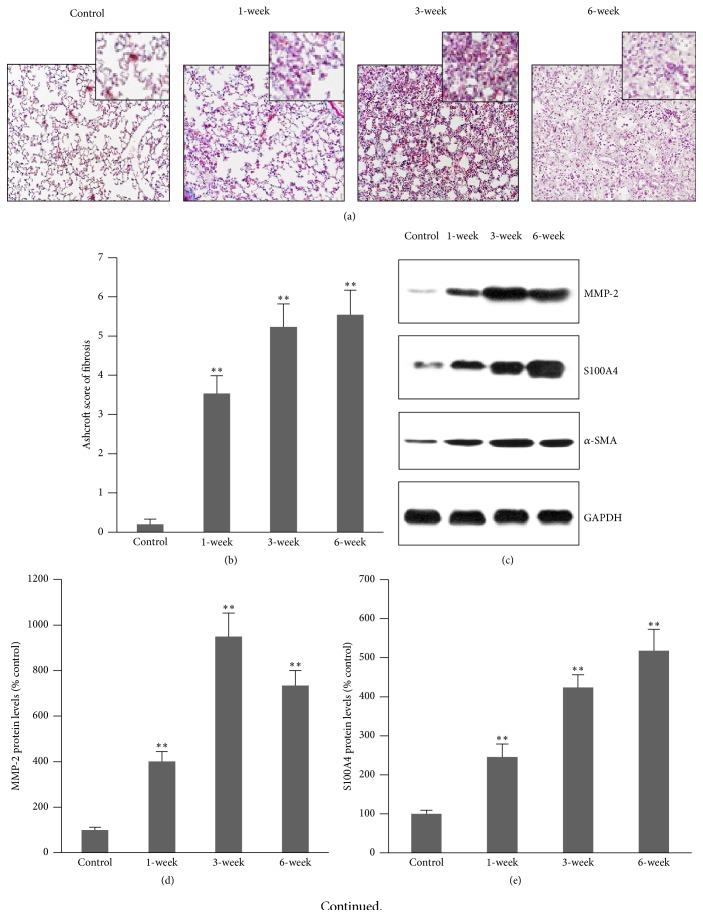
Bleomycin induces lung fibrosis. (a) A modified Masson trichrome staining. Alveolar septa thickening and cellular infiltration were observed in the lung tissues at 1 week after intratracheal bleomycin injection. (b) Quantitative analysis of fibrosis with Ashcroft scale. (c) Representative Western blots of MMP-2, S100A4, and *α*-SMA protein. Obvious increases in MMP-2, S100A4, and *α*-SMA protein expression were observed at 1-week, and progressive increase was observed from 1 to 6 weeks. GAPDH was used as a loading control. (d) Semiquantitative MMP-2 expression from densitometry analysis of bands in (c). (e) Semiquantitative S100A4 expression in (c). (f) Semiquantitative *α*-SMA expression in (c) (*n* = 10). ^*∗∗*^ *P* < 0.001* versus* control. *N* = 10.

The degree of pulmonary fibrosis was evaluated by H&E (not shown) and modified Masson's trichrome staining (Figure 1(a)). The peribronchial lesions with prominent alveolar septa thickening, cellular infiltrates, and single fibrotic mass were observed in lung tissues 1 week after intratracheal bleomycin injection. The single fibrotic masses became confluent, lung structure was severely damaged, and variable alveolar septa were mostly nonexistent at 3 weeks, and the lesions were consistently observed at 6 weeks after intratracheal bleomycin injection (Figure 1(a)). Fibrosis quantification was performed using a modified Ashcroft scale, and significant fibrosis was observed beginning at 1 week after bleomycin treatment (Figure 1(b)). MMP-2 protein level was previously reported to be increased in the lung tissues of bleomycin-treated animals and is preferentially secreted by fibroblasts and epithelial cells [8]. The protein level of MMP-2 in lung tissue homogenate was detected by Western blot (Figure 1(c)). A time-dependent increase in MMP-2 level was observed (Figure 1(d)). FSP1/S100A4^+^ cells are the primary fibroblasts in the lung [9]. Western blot showed that the S100A4 level progressively increased after intratracheal bleomycin injection (Figures 1(c) and 1(e)). Alpha-SMA is a myofibroblast marker [10]. Western blot showed that *α*-SMA level was also progressively increased after bleomycin injection (Figures 1(c) and 1(f)).

### 3.2. Epithelial-Mesenchymal Transition (EMT) in Lung Tissues

**Figure 2:**
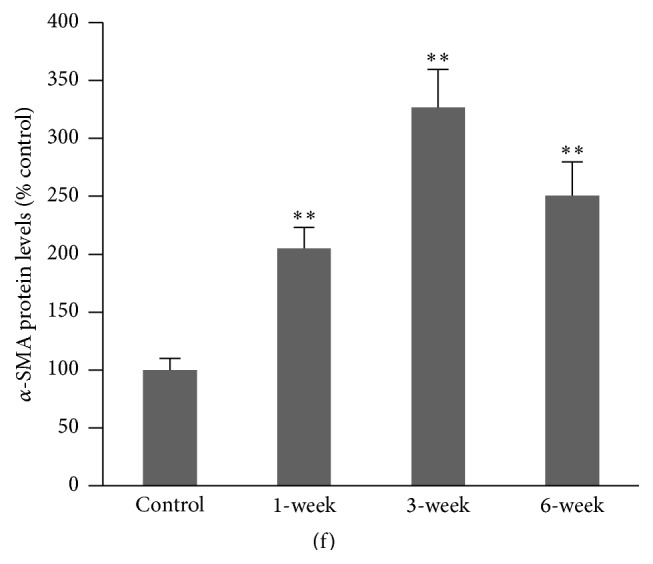
Protein levels of E-cadherin and vimentin in lung tissues of mice administered with bleomycin. The lung tissues of C57BL/6J mice were collected 1, 3, and 6 weeks following intratracheal bleomycin or saline instillation. (a) Immunofluorescence staining of E-cadherin and vimentin expression in lung tissue sections. Positive E-cadherin and vimentin staining was observed at the distal alveoli. (b) Representative Western blots of E-cadherin, N-cadherin, and vimentin protein in lung tissues. (c) Semiquantitative assay of Western blots of E-cadherin protein and vimentin protein expression in lung tissues. ^##^ *P* < 0.01, ^*∗*^ *P* < 0.05, and ^*∗∗*^ *P* < 0.001* versus* control. *N* = 10.

We measured E-cadherin and vimentin protein expression in the lung tissues of mice treated with bleomycin. Immunofluorescence staining showed that positive E-cadherin and vimentin staining cells can be observed at the distal alveoli (Figure 2(a)). Bleomycin administration significantly decreased the number of cells staining positively for E-cadherin, whereas an increased number of cells staining positively for vimentin were observed 3 and 6 weeks after bleomycin administration (Figure 2(a)). Similar results in E-cadherin and vimentin expression were observed in the Western blot analysis (Figures 2(b) and 2(c)). In contrast, no change in N-cadherin protein level was observed (Figure 2(b)). The observed changes in E-cadherin and vimentin expression may suggest the activation of EMT. 

### 3.3. Changes in Signal Molecules Associated with Hypoxia and Cell Differentiation

**Figure 3:**
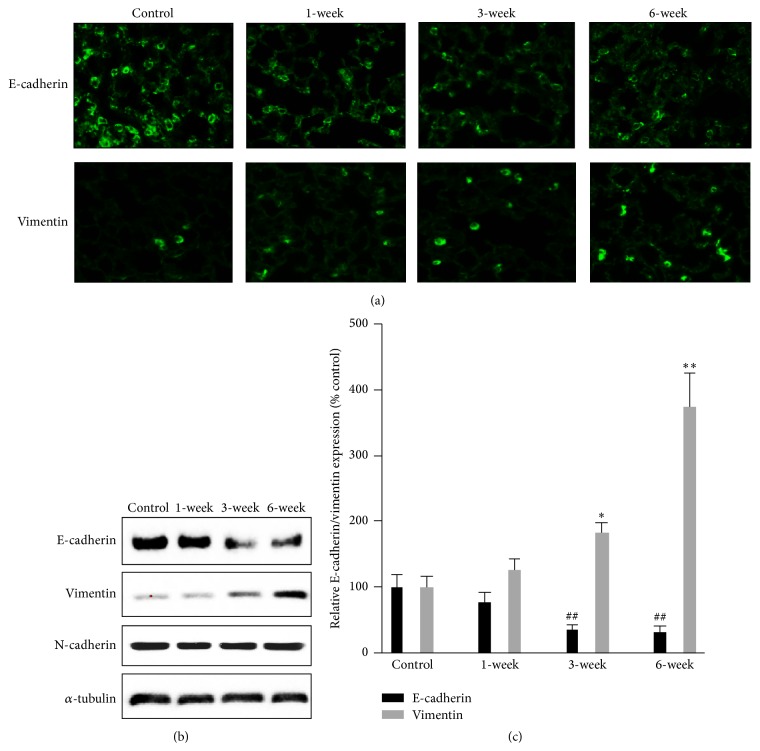
Protein expression of signal molecules in lung tissues. (a) Representative Western blots of protein expression in lung tissues. (b) Semiquantitative HIF-1*α* expression from densitometry analysis of bands in lung tissues. ^*∗∗*^ *P* < 0.001* versus *control; *n* = 10. (c) Semiquantitative ZEB1 expression in lung tissues. ^*∗∗*^ *P* < 0.001* versus* control; *n* = 10. (d) Semiquantitative phospho-p44/42 protein expression in lung tissues. ^*∗∗*^ *P* < 0.001* versus* control; *n* = 10. (e) Semiquantitative phospho-p38 MAPK protein expression in lung tissues. ^*∗*^ *P* < 0.01, ^*∗∗*^ *P* < 0.001* versus* control; *n* = 10. (f) Semiquantitative assay of Western blots of CD44 protein expression in lung tissues. (g) Immunohistochemical staining of CD44 protein. CD44 positive cells were obviously increased in alveoli in mice that received intratracheal bleomycin administration 1 week after treatment.

Pulmonary fibrosis often leads to severe hypoxia in lung tissues, and previous studies have observed the elevation of HIF-1*α* expression in lung fibroblasts exposed to bleomycin [23]. In this study, Western blot showed that the HIF-1*α* level in whole lung tissue homogenate was significantly increased 1 week after intratracheal bleomycin injection (Figures 3(a) and 3(b)). A recent study demonstrated that HIF-1*α* directly regulates ZEB1 expression, which subsequently activates EMT [20]. We further measured ZEB1 protein expression by Western blot and found that ZEB1 protein level was significantly increased in lung tissues 1–6 weeks after intratracheal bleomycin injection (Figures 3(a) and 3(c)). To explore what signaling is involved in the differentiation of EMT cells, we measured total p44/42, phospho-p44/42 (p-p44/42) (Figures 3(a) and 3(d)), total p38, and phospho-p38 MAPK (p-p38) (Figures 3(a) and 3(e)) protein levels in lung tissues. The phospho-p44/42 and phospho-p38 MAPK protein levels were time-dependently increased. In contrast, the total p44/42 and total p38 MAPK protein levels showed no change after bleomycin treatment. To investigate whether bleomycin-induced fibrosis is associated with the epithelial-mesenchymal transition, CD44^+^ expression was detected in lung tissues of mice injected with bleomycin intratracheally. Western blot showed that CD44^+^ protein level was significantly increased 1–6 weeks after bleomycin treatment (Figures 3(a) and 3(f)). Immunohistochemistry further showed that the CD44^+^ cells were significantly increased in mice that received intratracheal bleomycin injection 1 week after treatment compared to control mice (Figure 3(g)).

### 3.4. Epithelial-Mesenchymal Transition (EMT) and Associated Signaling* In Vitro*

**Figure 4:**
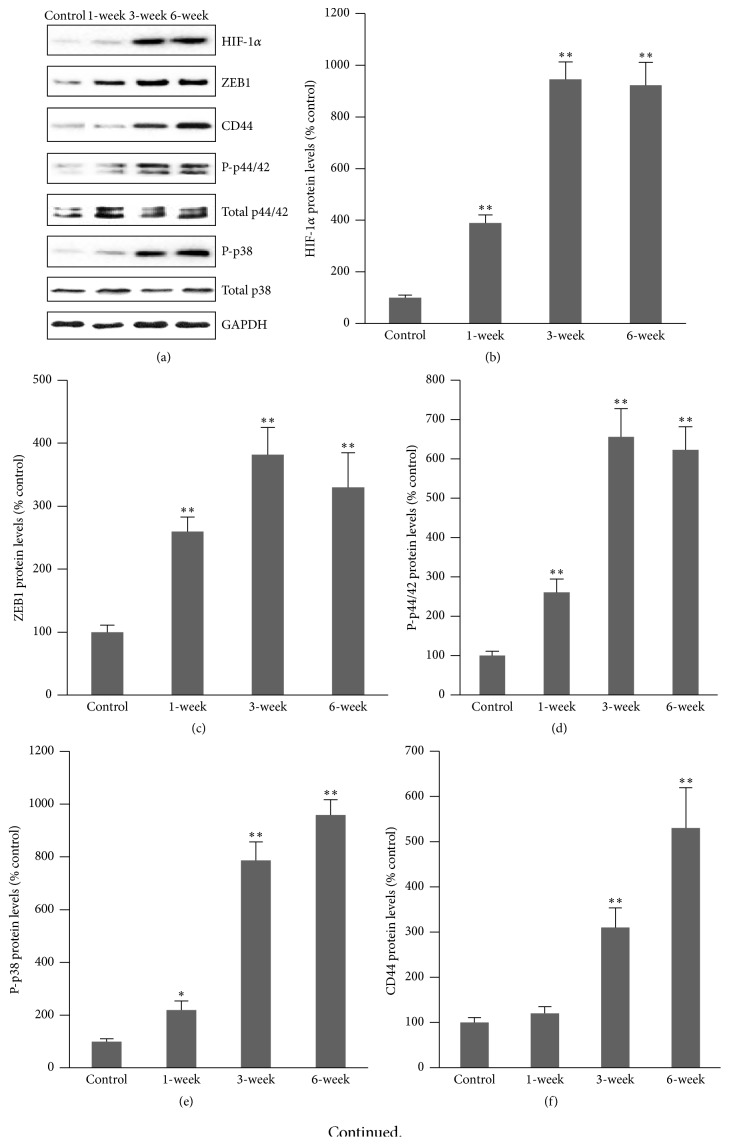
The evidence of epithelial–mesenchymal transition in cells. H358 cells were treated with hypoxia for 12 h (H-12 h) and 24 h (H-24 h). Control cells were grew up in normal oxygen. (a) Representative Western blots of HIF-1*α*, ZEB1, and EMT markers in H358 cells. (b) Semiquantitative assay of protein expression in lung tissues in (a). (c) Immunofluorescence staining of S100A4 protein. H358 and Beas-2B cells were treated with 2% O_2_ for 24 h with or without continuous treatment for 5 days with 10% O_2_. (d) Representative Western blots of CD44, phospho-p44/42, total p44/42, phospho-p38, and total p38 MAPK in H358 cells. GAPDH was used as a loading control. (e) Semiquantitative assay of protein expression in lung tissues in (c); *N* = 4.

To further validate the activation of EMT and explore its mechanisms, H358 cells were cultured under normoxic (21% O_2_) and hypoxic (2% O_2_) conditions for 12 h and 24 h. Hypoxia significantly increased HIF-1*α* and ZEB1 protein expression, decreased E-cadherin protein expression, and increased the protein expression of fibronectin and vimentin in H358 cells. No changes were observed in N-cadherin expression. The effects of hypoxia were more obvious at 24 h than at 12 h (Figures 4(a) and 4(b)). To investigate whether cells obtained the differentiation ability from EMT, H358 and Beas-2B cells were cultured under hypoxic (2% O_2_) conditions for 24 h and subsequently cultured under a mild hypoxic condition (10% O_2_) for 5 days. The expression of S100A4 protein was obviously increased at 24 h in H358 cells, and the increase was more significant after cells were cultured under a milder hypoxic condition (Figure 4(c)). In contrast, the increase in S100A4 protein expression was not obvious in Beas-2B cells comparing with H358 cells (Figure 3(c)). We therefore investigated the differentiation-associated signaling in H358 cells. Hypoxia at 2% O_2_ for 24 h significantly increased CD44 protein expression, p-p44/42, and p-p38 levels in H358 cells and subsequent treatment with 10% O_2_ caused more significant increase in CD44, p-p44/42, and p-p38 protein levels (Figures 4(d) and 4(e)). In contrast, no obvious changes in total p44/42 and total p38 MAPK protein levels were observed between different treatments (Figure 4(d)).

## 4. Discussion

This study demonstrated that intratracheal administration of bleomycin induced progressive lung fibrosis, which is related to progressive activation of EMT. Further molecular investigation in fibrotic lungs and hypoxic cells observed elevated protein levels of HIF-1*α* and ZEB1, which correlated with the activation of EMT. Importantly, increased p38 MAPK and Erk1/2 protein phosphorylation and the expression of CD44^+^ cells were observed in fibrotic lung tissues. The* in vitro* study validated the idea that pulmonary epithelial cells can gain the ability to differentiate into CD44^+^ cells under hypoxic conditions through the EMT mechanism. This study suggests that activation of EMT through HIF-1*α*-ZEB1 pathway is associated with progressive lung fibrosis induced by bleomycin.

MMP-2 is preferentially secreted by fibroblasts and epithelial cells and has been suggested to contribute to ECM deposition in patients with pulmonary fibrosis [8]. FSP1/S100A4^+^ cells are the primary fibroblasts in the lung [24] while *α*-SMA is a marker of myofibroblasts [10]. In this study, significant increase in lung fibrosis was observed 1 week after bleomycin administration, which parallels the increase of MMP-2, S100A4, and *α*-SMA protein levels. This suggests that progressive lung fibrosis was successfully established using a single dose of bleomycin injected intratracheally and that MMP-2 in fibroblasts is involved in the ECM deposition in lung.

This study revealed that E-cadherin protein expression was decreased, while vimentin was increased, in fibrotic lung tissues. Histological staining further showed the decrease in E-cadherin staining and the increase in vimentin and CD44 staining in distal alveoli 1 week after bleomycin treatment. CD44 protein level in whole lung tissue homogenate showed a similar tendency as changes in the amount of CD44^+^ cells in distal alveoli. CD44 is a marker for cells that acquired the differentiation ability from the EMT process [14]. Consistent with previous reports, this study provided solid evidence for the activation of EMT during the progression of bleomycin-induced fibrosis.

Although there is abundant evidence supporting the activation of EMT following major lung injury in mice, including bleomycin-induced fibrosis, the molecular mechanisms associated with the initiation of EMT have not been elucidated. A previous* in vitro* study demonstrated that hypoxia can induce EMT in cultured lung epithelial cells [25], but the study provided no further evidence for the signaling associated with EMT. Another* in vitro* study demonstrated that hypoxia induces EMT of alveolar epithelial cells through activation of mitochondrial HIF [18]. In this study, we also observed that hypoxia can activate EMT in type II pneumocytes through elevating HIF-1*α* protein levels. Previous studies in tumor cells revealed that a HIF-1*α*-ZEB1 signaling is involved in hypoxia-induced EMT [19, 20]. Our study observed elevation of HIF-1*α* and ZEB1 protein levels in both the fibrotic lung tissues* in vivo* and hypoxic pneumocytes* in vitro*, which correlated with EMT activation and lung fibrosis. Thus, our study first suggests that the HIF-1*α*-ZEB1-EMT pathway is involved in pulmonary fibrosis.

Although our and others' studies suggest that lung epithelial cells can acquire the ability to differentiate during EMT [14], the roles of EMT in fibrosis development and progression remain to be fully elucidated. A previous study in a repetitive bleomycin model observed that one-half of the S100A4^+^ fibroblasts were derived via EMT [24]. However, that observation was based on* in situ* immunolocalization of cells bearing a lineage trace of epithelial origin with a mesenchymal marker and may or may not have identified true myofibroblasts. In this study, hypoxia significantly increased S100A4 protein expression in hypoxic H358 cells. These changes were more obvious in H358 cells treated continuously with mild hypoxia. This is direct evidence for the hypoxia-induced EMT and differentiation of cells into fibroblasts. In contrast, hypoxia was less effective in stimulating S100A4 protein expression in Beas-2B cells. Interestingly, elevated expression of CD44 protein correlated with elevated p-p44/42 and p-p38 MAPK levels in H358 cells, suggesting that MAPK signaling is involved in the hypoxia-induced EMT and further differentiation of EMT cells into fibroblasts. TGF-*β*1 is a critical cytokine involved in the progression of fibrosis and a major inducer of EMT [26]. Previous studies have demonstrated that TGF-*β*1 can induce EMT in alveolar epithelial cells [27] and increase the expression of the myofibroblast marker *α*-SMA in injured lungs [28]. This study showed significant increases in *α*-SMA expression 1 week after bleomycin injection. Although TGF-*β*1 was not measured directly in this study, hypoxia has been widely demonstrated to induce endogenous TGF-*β*1 signaling [18]. Thus, multiple signaling pathways may be involved in EMT during lung injury.

In conclusion, bleomycin induces progressive lung fibrosis, which may be associated with the activation of EMT. The fibrosis-induced hypoxia may further activate EMT in distal alveoli through a hypoxia-HIF-1*α*-ZEB1 pathway and promote the differentiation of lung epithelial cells into fibroblasts through phosphorylation of p38 MAPK and Erk1/2 proteins.

## References

[1] Yarnold J., Brotons M.-C. V. (2010). Pathogenetic mechanisms in radiation fibrosis. *Radiotherapy and Oncology*.

[2] Lee J. C., Ahya V. N. (2010). Lung transplantation in autoimmune diseases. *Clinics in Chest Medicine*.

[3] Cohen R. A. C., Patel A., Green F. H. Y. (2008). Lung disease caused by exposure to coal mine and silica dust. *Seminars in Respiratory and Critical Care Medicine*.

[4] Ding N.-H., Li J. J., Sun L.-Q. (2013). Molecular mechanisms and treatment of radiation-induced lung fibrosis. *Current Drug Targets*.

[5] Daba M. H., El-Tahir K. E., Al-Arifi M. N., Gubara O. A. (2004). Drug-induced pulmonary fibrosis. *Saudi Medical Journal*.

[6] Hardie W. D., Le Cras T. D., Jiang K., Tichelaar J. W., Azhar M., Korfhagen T. R. (2004). Conditional expression of transforming growth factor-*α* in adult mouse lung causes pulmonary fibrosis. *American Journal of Physiology—Lung Cellular and Molecular Physiology*.

[7] Hirota N., Martin J. G. (2013). Mechanisms of airway remodeling. *Chest*.

[8] Corbel M., Caulet-Maugendre S., Germain N., Molet S., Lagente V., Boichot E. (2001). Inhibition of bleomycin-induced pulmonary fibrosis in mice by the matrix metalloproteinase inhibitor batimastat. *Journal of Pathology*.

[9] Zhang J., Chen L., Liu X., Kammertoens T., Blankenstein T., Qin Z. (2013). Fibroblast-specific protein 1/S100A4-positive cells prevent carcinoma through collagen production and encapsulation of carcinogens. *Cancer Research*.

[10] Zhang H.-Y., Gharaee-Kermani M., Zhang K., Karmiol S., Phan S. H. (1996). Lung fibroblast *α*-smooth muscle actin expression and contractile phenotype in bleomycin-induced pulmonary fibrosis. *American Journal of Pathology*.

[11] Wynn T. A. (2008). Cellular and molecular mechanisms of fibrosis. *Journal of Pathology*.

[12] Vaughan A. E., Chapman H. A. (2013). Regenerative activity of the lung after epithelial injury. *Biochimica et Biophysica Acta*.

[13] Tanjore H., Xu X. C., Polosukhin V. V. (2009). Contribution of epithelial-derived fibroblasts to bleomycin-induced lung fibrosis. *American Journal of Respiratory and Critical Care Medicine*.

[14] Lau A. N., Goodwin M., Kim C. F., Weiss D. J. (2012). Stem cells and regenerative medicine in lung biology and diseases. *Molecular Therapy*.

[15] Lin C.-Y., Peng C.-Y., Huang T.-T. (2012). Exacerbation of oxidative stress-induced cell death and differentiation in induced pluripotent stem cells lacking heme oxygenase-1. *Stem Cells and Development*.

[16] Park S., Ahn J.-Y., Lim M.-J. (2010). Sustained expression of NADPH oxidase 4 by p38 MAPK-AKT signaling potentiates radiation-induced differentiation of lung fibroblasts. *Journal of Molecular Medicine*.

[17] Tuder R. M., Yun J. H., Bhunia A., Fijalkowska I. (2007). Hypoxia and chronic lung disease. *Journal of Molecular Medicine*.

[18] Zhou G., Dada L. A., Wu M. (2009). Hypoxia-induced alveolar epithelial-mesenchymal transition requires mitochondrial ROS and hypoxia-inducible factor 1. *American Journal of Physiology—Lung Cellular and Molecular Physiology*.

[19] Joseph J. V., Conroy S., Pavlov K. (2015). Hypoxia enhances migration and invasion in glioblastoma by promoting a mesenchymal shift mediated by the HIF1*α*-ZEB1 axis. *Cancer Letters*.

[20] Zhang W., Shi X., Peng Y. (2015). HIF-1*α* promotes epithelial-mesenchymal transition and metastasis through direct regulation of ZEB1 in colorectal cancer. *PLOS ONE*.

[21] Hübner R.-H., Gitter W., El Mokhtari N. E. (2008). Standardized quantification of pulmonary fibrosis in histological samples. *BioTechniques*.

[22] Shi Y., Tohyama Y., Kadono T. (2006). Protein-tyrosine kinase Syk is required for pathogen engulfment in complement-mediated phagocytosis. *Blood*.

[23] Lu Y., Azad N., Wang L. (2010). Phosphatidylinositol-3-kinase/Akt regulates bleomycin-induced fibroblast proliferation and collagen production. *American Journal of Respiratory Cell and Molecular Biology*.

[24] Degryse A. L., Tanjore H., Xu X. C. (2010). Repetitive intratracheal bleomycin models several features of idiopathic pulmonary fibrosis. *American Journal of Physiology—Lung Cellular and Molecular Physiology*.

[25] Nurwidya F., Takahashi F., Kobayashi I. (2014). Treatment with insulin-like growth factor 1 receptor inhibitor reverses hypoxia-induced epithelial-mesenchymal transition in non-small cell lung cancer. *Biochemical and Biophysical Research Communications*.

[26] Willis B. C., Borok Z. (2007). TGF-*β*-induced EMT: mechanisms and implications for fibrotic lung disease. *American Journal of Physiology: Lung Cellular and Molecular Physiology*.

[27] Willis B. C., Liebler J. M., Luby-Phelps K. (2005). Induction of epithelial-mesenchymal transition in alveolar epithelial cells by transforming growth factor-*β*1: potential role in idiopathic pulmonary fibrosis. *The American Journal of Pathology*.

[28] Kim K. K., Kugler M. C., Wolters P. J. (2006). Alveolar epithelial cell mesenchymal transition develops in vivo during pulmonary fibrosis and is regulated by the extracellular matrix. *Proceedings of the National Academy of Sciences of the United States of America*.

